# Digestibility of diets containing calcium salts of fatty acids or soybean oil in horses^1^

**DOI:** 10.1093/tas/txaa001

**Published:** 2020-01-07

**Authors:** Laura K Fehlberg, James M Lattimer, Christopher I Vahl, James S Drouillard, Teresa L Douthit

**Affiliations:** 1 Department of Animal Sciences & Industry, Kansas State University, Manhattan, KS; 2 Department of Statistics, Kansas State University, Manhattan, KS

**Keywords:** cecum, digestibility, equine, lipid

## Abstract

Calcium salts of fatty acids (CSFAs) frequently are fed to ruminants, but their fate in the equine digestive system is unknown. The purpose of this study was to compare Enertia s/f, a proprietary CSFAs, and soybean (SB) oil with respect to impact on apparent total tract nutrient digestion and cecal fermentation parameters in horses. Eight cecally cannulated Quarter Horses were used in a crossover design in which horses consumed a diet for 32 d consisting of 1.5% body weight (BW) (as-fed) smooth bromegrass hay and 0.5% BW (as-fed) pelleted concentrate containing 4.9% CSFAs or 4.1% SB oil. Fecal samples were collected every 4 h from day 30 to 32 of each period and analyzed for apparent total tract digestibilities of dry matter (DM), neutral detergent fiber (NDF), acid detergent fiber (ADF), crude protein (CP), crude fat (CF), and gross energy (GE) using acid detergent insoluble ash as an internal marker. Cecal digesta was obtained at 0, 2, 4, 6, 8, 10, and 12 h following the morning meal on day 29 of each period and analyzed for pH and concentrations of volatile fatty acid (VFA) and long-chain fatty acids (LCFAs). Serum was collected on day 33 of each period following a 16-h fast and analyzed for triglycerides and cholesterol. Apparent total tract digestibilities of DM, NDF, ADF, CP, CF, and GE were unaffected by lipid source (*P* > 0.10). Serum triglycerides tended to be greater in horses consuming CSFAs compared to SB (*P* = 0.10); however, serum cholesterol was not different (*P* = 0.45). In horses consuming SB, cecal pH decreased below baseline (hour 0) at hours 2, 4, and 6 (*P* < 0.01), whereas cecal pH in horses consuming CSFAs was below baseline at hours 4 and 6 (*P* < 0.01). There were no treatment or time effects on cecal pH (*P* > 0.10). Cecal concentrations of total VFAs were greater in horses consuming SB compared to CSFAs at hour 2 (*P* = 0.01). Cecal concentrations of acetate, propionate, acetate:propionate (A:P), and butyrate were affected by time (*P* < 0.01). Propionate was less at hour 2 in horses fed CSFAs compared to horses fed SB (*P* = 0.04). A treatment × time interaction was detected for total cecal LCFAs concentration (*P* < 0.01); LCFAs concentration was greater at hour 2 for horses consuming CSFAs compared to horses fed SB (*P* = 0.02). SB oil and CSFAs have similar effects on the digestion of DM, NDF, ADF, GE, CF, and CP.

## INTRODUCTION

The gastrointestinal tract (GIT) of horses evolved to accommodate continuous grazing. Many performance horses have greater energy requirements than what can be met by forages alone; therefore, concentrates are often added to diets to increase energy density. Unfortunately, large amounts of dietary concentrates have the potential to cause digestive upset (Julliand et al., 2006). Fat can be used as a replacement for nonstructural carbohydrates (NSC) to safely increase energy density in equine diets (Kane et al., 1979). In ruminants, >6% ether extract (EE) in the diet depresses fiber fermentation (Palmquist and Jenkins, 1980; Doreau and Chilliard, 1997). Deleterious effects of fat on fiber digestibility in the rumen are due to the presence of a free carboxyl group on fatty acids; therefore, it was proposed that addition of a different functional group, such as Ca, may decrease lipid attachment to microflora and feed particles (Czerkawski et al., 1966). This causes the fat to become inert in the rumen but still allows absorption of the fat in the small intestine. In the equine, calcium salts of fatty acids (CSFAs) would dissociate into Ca and free fatty acids (FFAs) in the stomach and jejunum and then be absorbed in the duodenum, similarly to what is observed in the bovine; however, this has yet to be verified in the horse.

The amount of liquid fat added to animal diets often is limited by pellet durability and palatability (Casals et al., 2006). In concentrates, >2% to 3% added fat may result in a friable pellet (Partridge et al., 1986). Post-pellet liquid application (PPLA) of fat is possible, but no more than 15% fat may be added because of decreased palatability in horses (Hallebeek and Beynen, 2002). Also, PPLA is not available in many commercial feed mills. There have been claims from commercial feed manufacturers that replacing traditional fat supplements with CSFAs increases pellet durability and does not result in decreased palatability. For this reason, CSFAs may be a viable option to increase fat inclusion because it is a dry ingredient and can be added pre-pelleting.

Digestibility of diets containing CSFAs in horses is unknown, so the purpose of this study was to compare the influence of a proprietary CSFAs to soybean (SB) oil, a common source of supplemental fat in equine diets, on digestibility of dry matter (DM), organic matter (OM), neutral detergent fiber (NDF), acid detergent fiber (ADF), crude protein (CP), crude fat (CF), and gross energy (GE) in horses.

## MATERIALS AND METHODS

### Animals

All animal procedures were approved by the Institutional Animal Care and Use Committee at Kansas State University. Eight Quarter Horses (four mares, four geldings) with a mean BW of 535 ± 26.2 kg were used. All horses were fitted with a permanent cecal cannula (flexible ruminal cannula, #7c; 3.8 cm center diameter and 8.9 cm wall thickness; Bar Diamond, Parma, ID; Beard et al., 2011). Horses were housed in an enclosed facility in randomly assigned individual stalls (3.05 m × 3.66 m) bedded with pine shavings and had ad libitum access to water. All horses received 5 h to 6 h turnout per day in a drylot. There were four horses per drylot to accommodate socialization and exercise.

### Experimental Design

Horses were blocked by weight and sex and assigned to one of two initial treatments. Treatments were formulated to be isocaloric and isonitrogenous and consisted of concentrates containing either 4.1% SB oil or 4.9% CSFAs. CSFAs were supplied by Archer Daniels Midland Animal Nutrition (Quincy, IL) in the form of Enertia s/f and were provided in a pelleted concentrate. The CSFAs, which had less CF (84%) than SB (100%), was added at a level calculated to bring CF in both concentrates to 4.1%. Diets consisted of 1.5% BW smooth bromegrass hay and 0.5% BW pelleted concentrate (as-fed basis), divided equally between two meals fed at 0700 and 1900 hours (Tables 1, 2, and 3). Horses were maintained on their assigned treatment diets for a 28-d acclimation period followed by a 5-d collection period. Horses then were crossed over to the opposite treatment and the 33-d regimen was repeated.

**Table 1.** Proximate analysis (dry matter basis) of smooth bromegrass hay and pelleted concentrates containing SB oil or CSFAs

Item	Smooth bromegrass hay^1^	SB Concentrate^2^	CSFAs Concentrate^2,3^
Dry matter, %	92.2	89.6	90.1
Neutral detergent fiber, %	63.3	37.9	39.8
Acid detergent fiber, %	35.6	22.3	23.0
Crude protein, %	9.6	15.4	15.3
Crude Fat, %	2.7	7.9	6.8
Digestible energy, Mcal/kg	2.12	3.04	2.93

^1^Fed 1.5 % BW (as-fed) to all horses.

^2^Fed 0.5% of BW (as-fed) to horses in each respective treatment group (SB or CSFAs).

^3^CSFAs provided as Enertia s/f supplied by Archer Daniels Midland Animal Nutrition (Quincy, IL).

**Table 2.** Composition (DM basis) of pelleted concentrates containing either SB oil or CSFAs

Ingredient, %	SB concentrate	CSFAs concentrate^1^
Corn	9.09	9.84
Soybean hulls	30.56	30.61
Alfalfa meal	10.50	10.50
CSFAs	0.00	4.94
Soybean oil	4.17	0.00
Wheat middlings	36.73	36.25
Molasses	4.00	4.00
Soybean meal, 48%	2.13	2.15
Limestone	1.76	0.63
Sodium chloride	1.00	1.00
Copper sulfate	0.02	0.02
Zinc oxide	0.02	0.02
Vitamin A 30,000, IU/g	0.02	0.02

^1^CSFAs provided as Enertia s/f supplied by Archer Daniels Midland Animal Nutrition (Quincy, IL).

**Table 3.** Saturated fatty acid (SFA), mono-unsaturated fatty acid (MUFA), and poly-unsaturated fatty acid (PUFA) composition of smooth bromegrass hay and pelleted concentrates containing SB or CSFAs

Individual fatty acid^1,2^	Smooth bromegrass hay	SB concentrate	CSFAs^3^ concentrate
SFA			
C8:0	0.12	0.01	0.05
C10:0	0.07	0.06	0.10
C12:0	3.07	0.09	0.21
C14:0	1.23	0.12	0.81
C15:0	0.25	0.07	0.11
C16:0	22.59	13.07	34.58
C17:0	0.45	0.18	0.21
C18:0	2.10	3.34	4.01
C20:0	2.06	0.37	0.44
C21:0	0.19	0.05	0.04
C22:0	2.44	0.37	0.24
C23:0	0.43	0.09	0.10
C24:0	2.94	0.24	0.25
MUFA			
C16:1 *c*-9	0.75	0.18	0.25
C18:1 *t*-11	0.13	0.00	0.00
C18:1 *c*-9	3.79	19.93	28.78
C18:1 *c*-11	0.53	1.41	0.98
C22:1 *c*-9	0.26	0.07	0.10
C24:1 *c*-15	0.00	0.05	0.07
PUFA			
C18:2 *c*-9, *c*-11 (CLA)	0.00	0.05	0.00
C18:2 *c*-9, *t*-11 (CLA)	0.07	0.00	0.01
C18:2 *t*-9, *t*-11 (9-CLA)	0.18	0.11	0.22
C18:2 *c*-9, *c*-12	19.18	52.24	24.83
C18:2 *t*-9, *t*-12	0.44	0.03	0.05
C18:2 *t*-10, *c*-12 (10-CLA)	0.00	0.00	0.01
C18:3n3	35.88	7.32	2.92
C20:2 *c*-11, *c*-14	0.07	0.10	0.15
C20:3 *c*-8, *c*-11, *c*-14	0.29	0.02	0.04
C22:2 *c*-13, *c*-16	0.16	0.01	0.02
C22:5 *c*-7, *c*-10, *c*-13, *c*-16, *c*-19	0.14	0.00	0.00
Total fatty acid	100	100	100

^1^Presented as % of total fatty acids.

^2^Nomenclature of individual fatty acids defined as number of carbons:number of double bonds. Numbers and letters represent placement of carbon atom and orientation on either side of the double bond described as *t = trans* or *c = cis*.

^3^CSFAs provided as Enertia s/f supplied by Archer Daniels Midland Animal Nutrition (Quincy, IL).

### Sample Collection

Cecal samples from all horses were collected 0, 2, 4, 6, 8, 10, and 12 h following the morning feeding on day 29 of each period. Approximately 50 mL cecal contents were obtained through cannulae via gravity flow, strained through four layers of cheesecloth, and collected into a container (Specimen Storage Containers, #14955117A, Fisher Scientific, Pittsburg, PA). Immediately after collection, pH of strained cecal fluid was measured using a portable pH meter (Accumet Portable pH Meter AP62, Fisher Scientific, Pittsburg, PA). One milliliter aliquots of strained cecal fluid from each horse for each time point were transferred in duplicate into microcentrifuge tubes. Deproteinization was achieved with 250 µL of 25% (wt/vol) meta-phosphoric acid solution and samples were stored at −18°C until volatile fatty acid (VFA) analysis. Twenty-five milliliters of remaining strained cecal fluid were transferred to a 25 mm × 150 mm glass screw-top tube with a Teflon lined cap (Fisher Scientific, Pittsburg, PA) and stored at −18°C until analysis of long-chain fatty acids (LCFAs). Following final cecal collection in each period, there was a 12-h buffer period where no collections were obtained. On day 30 of each period, bedding was removed from stalls. From day 30 to 32, feces were collected from stall floors every 6 h. Feces contaminated with cecal fluid or urine were discarded. Each day, feces from each horse were homogenized and a sample was transferred to a 3.8-liter Ziploc bag (S. C. Johnson and Son Inc., Racine, WI). Samples from each day were combined and homogenized into 1 sample for each horse (approximately 2.3 kg) and stored at −18°C for further analysis of DM, OM, NDF, ADF, CP, CF, and GE.

Following fecal collections, horses were fasted for 16 h on day 33 and approximately 15 to 20 mL whole blood was obtained via jugular venipuncture using a 38.1-mm, 20-gauge needle. Blood was collected into 10-mL non-heparinized red top Vacutainer serum tubes (#366430, Becton, Dickson and Company, Franklin Lakes, NJ), left at room temperature until a clot was identified, and centrifuged at 1,400 × g at room temperature for 20 min. Serum was removed using a transfer pipette and approximately 1 mL was deposited into 1.5-mL microcentrifuge tubes in quadruplicate. Tubes were stored at −18°C for future analysis of serum triglycerides (TGs) and cholesterol.

### Laboratory Analyses

Fecal samples were dried at 55°C in a forced-air oven for 48 h and ground using a Wiley mill (1-mm screen; Thomas Scientific, Philadelphia, PA). Samples from hay and concentrates were ground through a 1-mm screen using a Wiley mill. Subsamples (0.5 g) of feedstuffs and feces were dried at 105°C in a forced-air oven to determine DM. Separate 3-g subsamples of hay, concentrate, and feces were combusted in a muffle oven at 450°C for 8 h for quantification of ash and OM (Undersander et al., 1993). Dried feces, hay, and concentrate were analyzed for NDF and ADF using a batch processor while following the procedure outlined by Goering and van Soest (1970; Ankom Technology Corp., Fairport, NY). Following ADF analysis, bags were secured in tins, weighed, and combusted in a muffle oven at 450°C for 8 h to determine acid detergent insoluble ash (ADIA; Kanani et al., 2014). ADIA was calculated using the following equation:

$$\begin{array}{l} ADIA\left(\%\right)=100*\left[\frac{\begin{array}{c} Sample\;weight\\ \;after\;combustion\left(g\right) \end{array}}{Initial\;weight\;of\;sample\left(g\right)}\right] \end{array}$$

Adiabatic bomb calorimetry was used to determine GE in dried feed and feces (AOAC, 1990). The CF content of feed and feces was determined using acid hydrolysis where hydrochloric acid was utilized to liberate fat from calcium (AOAC, 2012a). Crude protein in feed and feces was determined using the combustion method to determine N content (g) and multiplying N by 6.25 (AOAC, 2012b). ADIA was used as an internal marker for the calculation of digestibility of DM, NDF, ADF, CP, CF, and GE. Digestibilities were calculated using the following equation:

$$\begin{array}{l} Digestibility\left(\%\right)=100*[1-\left(\frac{\begin{array}{c} nutrient\;in\\ \;feces\;\left(\%\right)\\ {}*ADIA\\ \;in\;feed\;\left(\%\;\right) \end{array}}{\begin{array}{c} nutrient\;in\\ \;feed\;\left(\%\right)\\ {}*ADIA\\ \;in\;feces\;\left(\%\;\right) \end{array}}\right)] \end{array}$$

Total dietary compositions were determined using this equation:

$$\begin{array}{ll} Nutrient\;in\;feed(\;\%\;)= & \left[nutrient\;in\;hay\;\left(\;\%\right)*0.75\right]\\ +\left[nutrient\;in\;grain\;(\;\%\;)*0.25\right] \end{array}$$

Following deproteinization, strained cecal samples were frozen for at least 24 h, later thawed, homogenized, and centrifuged at 17,000 × g for 15 min. The aqueous supernatant was transferred into 12 mm × 32 mm gas chromatography (GC) vials, vortexed (Scientific Industries Vortex-Genie 2, Houston, TX), and analyzed using a packed column (6’ × ¼”, 4 mm ID glass, packed with GP 10% SP-1200, 1% H_3_PO_4_; Supelco #1-1965; Agilent Technologies, Santa Clara, CA) with a flame ionization detector (compressed air set at 200 mL/min and H_2_ set at 20 mL/min). Nitrogen was used as the carrier gas with a flow rate of 60 mL/min. The detector and injector were set at 250°C and the column was at a constant temperature of 130°C. VFAs were quantified by comparing to known standards (Supelco Volatile Fatty Acid Standard Mix; Sigma-Aldrich, St. Louis, MO) containing acetate, propionate, isobutyrate, butyrate, isovalerate, and valerate.

For fatty acid analysis (C8 to C24), cecal samples were removed from a −18°C freezer and transferred directly to a precooled drying chamber of a lyophilizer (Genesis Model 35EL, SP Scientific; Warminster, PA) programmed with vacuum of 100 mTorr, shelf temperature of −10°C, and condenser chamber temperature of −70°C. After 48 h, samples were removed and interesterified to create methyl-esters (Sukhija et al., 1988). Interesterified samples were centrifuged at 500 × *g* for 10 min, and the upper organic solvent layer was transferred to screw-top 12 mm × 32 mm GC vials for GC. An SP-2560 capillary column (100 m × 0.25 mm with a 0.2 µm film; Agilent and J&W columns, Santa Clara, CA) with a flame ionization detector was used. Hydrogen was used as the carrier gas with a flow rate of 1 mL/min and a split ratio of 1:100. The injection and detector temperatures were set at 250°C. Initial oven temperature was 140°C. Temperature increased at 2°C/min until 200°C was reached, and this was followed by an increase of 4°C/min until a final oven temperature of 245°C was achieved. Fatty acids were quantified by comparing to known standards (Supelco 37 FAME mix #47885-U; Sigma-Aldrich, St. Louis, MO) containing C6:0, C8:0, C10:0, C11:0, C12:0, C14:0, C14:1, C15:0, C15:1, C16:0, C16:1, C17:0, C17:1, C18:0, C18:1n9t, C18:1n10t, C18:1n11t, C18:1n9c, C18:1n11c, C18:2n6t, C18:2n6c, C20:0, C18:3n6, C20:1, C18:3n3, CLA 9c11t, C21:0, CLA 10t12c, CLA 9c11c, CLA 9t11t, C20:2, C22:0, C20:3n6, C22:1n9, C20:3n3, C20:4n6, C23:0, C22:2, C24:0, C20:5n3, C24:1, C22:5n3, and C22:6n3.

Serum TGs and cholesterol were measured at the Animal Health and Diagnostic Center at Cornell University using the triglyceride GPO-PAP method and cholesterol CHOD-PAP method, respectively (Roche ModP, Roche Diagnostics GmbH, Sandhofer Strasse 116, D-68305 Mannheim; Roche Diagnostics, Indianapolis, IN).

### Statistical Analyses

Data were analyzed using the MIXED procedure of SAS (Version 9.4). For digestibility variables (DM, NDF, ADF, CP, CF, and GE) and serum concentrations of TGs and cholesterol, the model included fixed effect of treatment and random effects of replicate (horse) and period to determine the least-squares means (LSMEANS), and experimental unit was horse. For pH, VFAs, and LCFAs, a repeated measures statement was utilized and the model included fixed effect of treatment and treatment by hour interaction and random effect of replicate (horse) and period to determine LSMEANS. Linear and quadratic contrasts for seven equally spaced time points were included to determine hour trends. Repeated statement included horse as the subject, time, and autoregressive(1) as the covariance structure. The 101 Kenward–Rogers correction was applied for degrees of freedom estimation for all analyses. Significance was declared at *P* < 0.05, and a tendency was considered to be present when 0.05 < *P* < 0.10. Differences among LSMEANS were determined using the PDiff option of SAS. A Bonferroni adjustment was used to correct for multiplicity.

## RESULTS

Body weights were not different between initiation and conclusion of the project, nor were they affected by treatment (*P* > 0.10). DM intake was not different between treatments (*P* > 0.10), and both concentrates were consumed in their entirety within 30 min of feeding throughout the duration of the experiment. Apparent total tract digestibilities of DM, NDF, ADF, CP, CF, and GE were unaffected by lipid source (*P* > 0.10; Table 4). Concentrations of cholesterol in the serum were not different between horses fed either diet (*P* > 0.10). In contrast, concentrations of TGs in the serum tended to be greater in horses fed CSFAs compared to those consuming SB (*P* = 0.09; Table 5).

**Table 4.** Total tract digestibility coefficients of horses consuming diets supplemented with SB oil or CSFAs for 32 d^†^

Item^1^	SB	CSFAs^2^	SEM
Dry matter, %	41.42	42.44	5.85
Organic matter, %	41.13	42.11	3.95
Neutral detergent fiber, %	19.64	21.12	3.47
Acid detergent fiber, %	18.48	19.54	2.18
Crude protein, %	86.56	86.41	0.98
Crude fat, %	79.11	77.37	1.90
Gross energy, %	39.29	39.90	5.22

^†^Diets consisted of 1.5% BW smooth bromegrass hay (as-fed) and 0.5% BW pelleted concentrate (as-fed) containing either 4.1% SB or 4.9% CSFAs.

^1^Digestibility coefficients presented on a percentage basis calculated using acid detergent insoluble ash as an internal marker in feces collected on day 30 to 32.

**Table 5.** Serum^‡^ triglycerides and cholesterol in horses consuming diets supplemented with SB or CSFAs for 32 d^†^

Item	SB	CSFAs^1^	SEM
Serum triglycerides, mmol/L	1.14^A^	1.33^B^	0.11
Serum cholesterol, mmol/L	6.29	6.19	0.25

^‡^Serum was collected following a 16-h fast on day 33.

^†^Diets consisted of 1.5% BW smooth bromegrass hay (as-fed) and 0.5% BW pelleted concentrate (as-fed) containing 4.1% SB or 4.9% CSFAs.

^1^CSFAs provided as Enertia s/f supplied by Archer Daniels Midland Animal Nutrition (Quincy, IL).

^A, B^Indicates a difference between treatments at 0.05 < *P* < 0.10.

**Figure 1.**
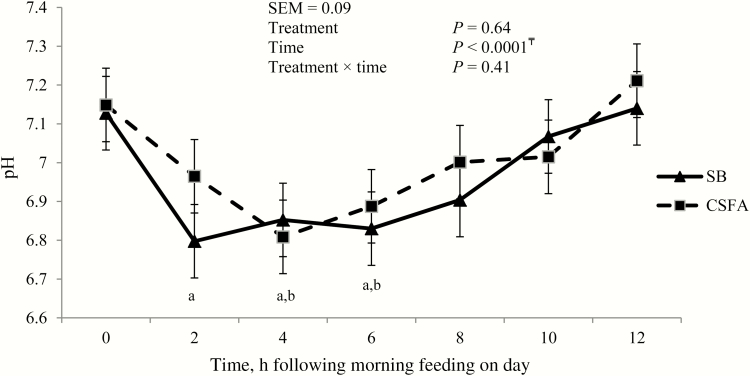
Cecal pH in horses consuming diets supplemented with SB oil or CSFAs^*^ for 32 d^†^.^†^Diets consisted of 1.5% BW smooth bromegrass hay (as-fed) and 0.5% BW pelleted concentrate (as-fed) containing 4.1% SB or 4.9% CSFAs. ^*^CSFAs provided as Enertia s/f supplied by Archer Daniels Midland Animal Nutrition (Quincy, IL). ^a^Indicates cecal pH below baseline (hour 0) value in horses consuming SB (*P* < 0.05). ^b^Indicates cecal pH below baseline (hour 0) value in horses consuming CSFAs (*P* < 0.05). ^₸^Indicates a quadratic effect of time averaged across treatments (*P* < 0.05).

There was an effect of time on cecal pH (*P* < 0.01; Figure 1). In horses consuming SB, cecal pH was below baseline (hour 0) at hours 2, 4, and 6 (*P* < 0.01) and was not different from baseline at hours 8, 10, and 12 (*P* > 0.10). Cecal pH of horses consuming CSFAs was below baseline at hours 4 and 6 (*P* < 0.01), but was not different from baseline at hours 8, 10, and 12 (*P* > 0.10). There were no treatment effects on cecal pH (*P* > 0.10).

There was a quadratic effect of time on total cecal VFAs concentration (*P* < 0.001; Table 6), as total cecal VFA concentrations were elevated above baseline at hours 2, 4, 6, 8, and 10 (*P* < 0.05) and were not different from baseline at hour 12 (*P* > 0.10) in horses consuming SB. In horses consuming CSFAs, total cecal VFA concentrations were above baseline at hours 4, 6, and 8 (*P* < 0.001) and were not different from baseline at hours 2, 10, and 12 (*P* > 0.10). Total cecal VFA concentrations tended to be greater in horses consuming SB compared to horses consuming CSFAs at hour 2 (*P* = 0.07). By hour 4, total cecal VFA concentrations were not different in horses consuming the two diets, which continued through hour 12 (*P* > 0.10).

**Table 6.** Cecal VFAs concentration in horses consuming diets supplemented with SB oil or CSFAs for 32 d^†^

VFAs, mM	Time	SB	CSFAs^1^	SEM	Fixed effects^2^ (*P* < 0.05)
Total VFAs	0	43.06^a^	39.28^acd^	3.86	T^3^
	2	51.25^C,b^	37.02^D,acd^		
	4	56.20^b^	57.38^b^		
	6	57.77^b^	55.73^be^		
	8	55.13^b^	51.72^be^		
	10	52.39^b^	45.53^cde^		
	12	43.50^a^	38.05^d^		
Acetate	0	31.70^a^	29.19^a^	2.77	T^3^
	2	38.05^C,b^	27.97^D,a^		
	4	41.64^b^	43.10^b^		
	6	42.71^b^	41.11^bc^		
	8	40.07^b^	38.27^bc^		
	10	38.19^b^	34.03^acd^		
	12	31.35^a^	28.21^ad^		
Propionate	0	8.37 ^a^	7.54^a^	0.86	T^3^
	2	9.76^A,ab^	6.53^B,a^		
	4	10.85^b^	10.80^b^		
	6	11.37^b^	11.18^b^		
	8	11.17^b^	10.23^b^		
	10	10.53^b^	8.67^ab^		
	12	8.95^ab^	7.45^a^		
Acetate:propionate	0	3.78^ab^	3.91^ac^	0.18	T^4^
	2	3.93^b^	4.32^b^		
	4	3.87^bc^	4.04^c^		
	6	3.77^ab^	3.72^a^		
	8	3.57^a^	3.76^ac^		
	10	3.68^ab^	3.96^ac^		
	12	3.56^ac^	3.81^ac^		
Butyrate	0	2.48^a^	2.14^ac^	0.37	T^3^
	2	2.85^ab^	2.02^a^		
	4	3.04^ab^	2.97^b^		
	6	3.18^ab^	3.03^b^		
	8	3.37^b^	2.85^c^		
	10	3.13^ab^	2.49^a^		
	12	2.70^ab^	2.05^a^		
Valerate	0	0.17^a^	0.13^ab^	0.04	T^3^
	2	0.20^ab^	0.16^ab^		
	4	0.22^b^	0.18^a^		
	6	0.18^ab^	0.16^ab^		
	8	0.18^ab^	0.15^ab^		
	10	0.19^ab^	0.13^ab^		
	12	0.17^ab^	0.11^b^		
Isobutyrate	0	0.21	0.17^ab^	0.05	T^3^
	2	0.24	0.24^a^		
	4	0.30	0.21^ab^		
	6	0.21	0.17^ab^		
	8	0.22	0.16^ab^		
	10	0.22	0.13^b^		
	12	0.21	0.16^ab^		

^†^Diet consisted of 1.5% BW smooth bromegrass hay (as-fed) and 0.5% BW pelleted concentrate (as-fed) containing 4.1% SB or 4.9% CSFAs.

^1^CSFAs provided as Enertia s/f supplied by Archer Daniels Midland Animal Nutrition (Quincy, IL).

^2^T = time effect, X = treatment effect, and I = treatment × time effect.

^3^Indicates a quadratic effect of time averaged across treatments (*P* < 0.05).

^4^Indicates a linear effect of time averaged across treatments (*P* < 0.05).

^A,B^Indicates a difference between treatments at *P* < 0.05.

^C,D^Indicates a difference between treatments at 0.05 < *P* < 0.10

^a,b,c,d,e^Indicates a difference within treatments at *P* < 0.05.

Cecal concentrations of acetate, propionate, and butyrate, as well as acetate:proprionate ratio (A:P), changed over time (*P* < 0.01; Table 6). Cecal acetate concentration was above baseline (hour 0) at hours 2, 4, 6, 8, and 10 (*P* < 0.05) in horses consuming SB. Elevation of cecal acetate concentration above baseline in horses consuming CSFAs occurred at hours 4, 6, and 8 (*P* < 0.01) and returned to baseline at hours 10 and 12 (*P* > 0.10). Cecal propionate concentrations were elevated above baseline at hours 4, 6, 8, and 10 (*P* < 0.03) in horses consuming SB and were elevated above baseline at hours 4, 6, and 8 in horses consuming CSFAs (*P* < 0.01). Horses consuming SB tended to have greater cecal acetate concentrations (*P* = 0.08) and greater propionate concentrations at hour 2 compared to those consuming CSFAs (*P* = 0.04). In horses consuming CSFAs, cecal A:P increased above baseline at hour 2 (*P* < 0.001); however, cecal A:P was not different from baseline in horses consuming SB at any time point (*P* > 0.10). Lipid source did not have an effect on A:P (*P* > 0.10). Cecal butyrate concentration was elevated above baseline at hours 4 and 6 in horses consuming CSFA and at hour 8 in horses consuming SB (*P* < 0.05); however, differences were not noted between horses consuming the two diets (*P* > 0.10). Cecal concentrations of valerate and isobutyrate were affected by time (*P* < 0.05). In horses consuming SB, cecal concentrations of valerate were increased at hour 2 (*P* < 0.01) compared to baseline, but not different at any other time point.

Treatment × time interactions were observed in cecal concentrations of total LCFAs, saturated LCFAs, and unsaturated LCFAs (*P* < 0.05; Table 7). Cecal concentrations of total LCFAs and unsaturated LCFAs were greater at hour 2 in horses consuming CSFAs compared to horses consuming SB (*P* < 0.05). Tendencies for increased cecal concentrations of saturated LCFAs were detected at hours 2 and 8 (*P* = 0.07) in horses consuming CSFAs compared to horses consuming SB. There was also a treatment × time interaction in cecal concentrations of palmitic acid (C16:0), oleic acid (C18:1n9c), and linoleic acid (C18:2n6c), with greater concentrations in horses consuming CSFAs compared to those consuming SB at hour 2 (*P* < 0.05). There was no observed effect of treatment (*P* > 0.05; data not shown) on cecal concentrations of conjugated linoleic acid (C18:2n9c11t), heneicosylic acid (C21:0), conjugated linoleic acid (C12:2n9c11c), behenic acid (C22:0), dihomogamma linolenic acid (C20;3n6), erucic acid (C22:1n9), alpha-linolenic acid (C20:3n3), arachidonic acid (C20:4n6), tricosylic acid (C23:0), docosadienoic acid (C22:2), lignoceric acid (C24:0), and nervonic acid (C23:1).

**Table 7.** Cecal LCFAs concentration in horses consuming diets supplemented with SBSB or CSFAs for 32 d^†^

LCFAs, µg/mL^1^	Time	SB	CSFAs^2^	SEM	Fixed effects^3^ (*P* < 0.05)
Total LCFAs	0	99.79^ab^	96.94^a^	17.56	T, I
	2	120.57^A,a^	183.23^B,b^		
	4	81.56^b^	110.22^a^		
	6	76.47^b^	107.73^a^		
	8	76.47^b^	112.57^a^		
	10	81.10^b^	99.55^a^		
	12	104.10^ab^	90.36^a^		
Total saturated LCFAs	0	62.63^abc^	62.09^a^	10.95	T, I
	2	70.95^C,ac^	101.68^D,b^		
	4	49.72^bc^	65.36^a^		
	6	44.85^b^	67.80^a^		
	8	43.54^C,b^	68.19^D,a^		
	10	50.02^ab^	61.18^a^		
	12	67.50^c^	53.95^a^		
Total unsaturated LCFAs	0	37.17^a^	34.85^a^	7.26	T, I
	2	49.62^A,b^	81.55^B,b^		
	4	31.83^a^	44.86^a^		
	6	31.35^a^	40.59^a^		
	8	32.76^a^	44.38^a^		
	10	31.08^a^	38.41^a^		
	12	36.60^a^	35.72^a^		
Palmitic acid (C16:0)	0	34.88^abcd^	33.04^a^	7.86	T, I
	2	43.65^A,acd^	65.49^B,b^		
	4	30.49^b^	41.73^a^		
	6	27.70^b^	45.18^a^		
	8	27.19^b^	44.71^a^		
	10	31.19^c^	37.49^a^		
	12	42.69^d^	29.76^a^		
Oleic acid (C18:1 *c*-9)	0	10.99^a^	9.97^a^	2.84	T, I
	2	17.76^A, b^	32.10^B, b^		
	4	10.53^a^	15.26^a^		
	6	9.36^a^	13.76^a^		
	8	9.42^a^	12.67^a^		
	10	9.58^a^	10.47^a^		
	12	11.61^ab^	8.86^a^		
Linoleic acid (C18:2 *c*-9, *c*-12)	0	7.82	7.47^a^	2.09	T, I
	2	10.19^A^	21.18^B, b^		
	4	7.43	9.56^a^		
	6	7.19	9.47^a^		
	8	7.34	8.88^a^		
	10	6.26	7.74^a^		
	12	7.48	7.37^a^		
Eicosapentaenoic acid (C22:5 *c*-7, *c*-10, *c*-13, *c*-16, *c*-19)	0	0.51^a^	0.54^a^	0.12	T, I
	2	0.35^ab^	0.38^ab^		
	4	0.15^b^	0.31^ab^		
	6 8	0.42^ab^ 0.33^ab^	0.26^b^ 0.43^a^		
	10	0.47^ab^	0.41^a^		
	12	0.83^A,c^	0.34^B,a^		

^†^Diet consisted of 1.5% BW smooth bromegrass hay (as-fed) and 0.5% BW pelleted concentrate (as-fed) containing 4.1% SB or 4.9% CSFAs.

^1^Nomenclature of individual fatty acids defined as number of carbons:number of double bonds. Numbers and letters represent placement of carbon atom and orientation on either side of the double bond described as *t* = *trans* or *c* = *cis*.

^2^CSFAs provided as Enertia s/f supplied by Archer Daniels Midland Animal Nutrition (Quincy, IL).

^3^T = time effect, X = treatment effect, and I = treatment × time effect.

^A,B^Indicates a difference between treatments at *P* < 0.05.

^C,D^Indicates a difference between treatments at 0.05 < *P* < 0.10

^a,b,c,d,e^Indicates a difference within treatments at *P* < 0.05.

## DISCUSSION

On day 17 of period 1, a horse consuming CSFAs presented with signs of colic. A veterinarian from the Veterinary Health Center at Kansas State University was consulted and a physical exam was completed. Large colon displacement was suspected; however, vital signs were within normal limits (temperature: 98.7°F; heart rate: 24 beats per min; respiratory rate: 16 breaths per min). Mineral oil and electrolytes were mixed with 18.9 liters of water and administered via nasogastric tube. Flunixin (1.03 mg/kg), xylazine (0.29 mg/kg), and butorphanol (0.01 mg/kg) were administered intravenously and feed was withheld for 12 h. Hay was slowly reintroduced starting 12 h after symptoms subsided and grain was reintroduced after 24 h. Because hindgut disturbances may have occurred, the initial feeding period was extended by 1 wk for all horses to allow normal motility and fermentation to be restored in the colic patient. The acclimation period of period 2 was also extended 1 wk; thus, both acclimation periods were 28 d rather than the originally planned 21 d. No other signs of colic were noted throughout the experiment; however, this horse was found deceased the morning following the conclusion of this experiment. Technicians at the Veterinary Health Center were unable to identify the cause of death through standard necropsy procedures. Data obtained from this horse were not outliers compared to other horses on study; therefore, data collected from this horse were included in the statistical analyses.

Although treatments were formulated to be iso-fat, proximate analysis revealed that SB concentrate was slightly greater in CF. This discrepancy could be attributed to analytical variation, improper mixing at the feed mill, or incomplete dissociation of Ca from the fatty acids.

This experiment was the first of its kind to evaluate the effect of CSFAs on nutrient digestibility in the horse. In dairy cows supplemented with 4.5% added soy fatty acids or soy calcium soaps, no differences in DM, NDF, ADF, CP, nor CF digestibilities were detected (Jenkins and Palmquist, 1984). Furthermore, digestibilities of DM, ADF, and NDF in dairy cows fed varying fat sources added at 6.8%, including an animal-vegetable blend and a palm oil calcium soap, were not different (Palmquist, 1991). Based on this experiment, it appears that supplementation with CSFAs does not alter digestion of most basic nutrients in the horse either, despite differences in gastrointestinal anatomy between the bovine and equine.

Though digestibilities of nutrients were not different between horses on the two diets in this study, the coefficients differ from what has been reported by others. Digestibility of DM may be slightly decreased in a cecally cannulated horse compared to an intact horse (Jansen et al., 2002), but this likely does not fully account for the large difference in DM digestibility noted in the current study compared with other studies. The poor quality and increased quantity of hay used in this experiment likely contributed to the decreased digestibility coefficients calculated. Delobel et al. (2008) included fat in diets of horses at a comparable level and observed a 20% greater DM digestibility than what was observed in the current study; however, their forage:concentrate was 50:50, whereas it was 75:25 in the current study. Increased bulk in the diet, such as from the long-stemmed hay provided in the current experiment, increases the rate of passage and decreases the time available to digest feedstuffs (Stevens and Hume, 1996), which may help explain the decreased digestibility observed in the current experiment. In this study, digestibility of OM was similar to that of DM, which is in accordance with what others have observed (Cuddeford and Hughes, 1990; Lindberg and Karlsson, 2001; Gatta et al., 2005). In the current study, NDF and ADF digestibilities were 20% to 30% less than what has been reported in the literature with similar fat supplementation levels in horses; however, dietary NDF, ADF, and forage:concentrate ratios provided in previous work were less than that utilized in the current study (Lindberg and Karlsson, 2001; Gatta et al., 2005; Delobel et al., 2008).

In contrast to what was noted for DM, NDF, and ADF, digestibility of CP in the present study was greater than what has been reported in previous work (West and Hill, 1990; Miraglia et al., 1999; De Marco et al., 2012). Delobel et al. (2008), however, supplemented 8% fat to horses fed a 50:50 forage:concentrate and noted a CP digestibility similar to that reported for the current study. It is difficult to compare digestibility of CP between studies because of variability in protein sources (Miraglia et al., 1999) and fiber content (De Marco et al., 2012). Digestibility of CF in the current study was similar to observations by previous researchers when fat was supplemented to horses (Jansen et al., 2002; Gatta et al., 2005; Delobel et al., 2008). Initially, fecal CF was analyzed in the current study using the EE method without acid hydrolysis. On second analysis, CF was determined via acid hydrolysis to liberate any Ca attachments that may still exist. Because both laboratory techniques yielded the same fecal EE content, it was concluded that fats in the feces were not in the form of Ca soaps.

Serum was collected for the measurement of TGs and cholesterol after a 16-h fast in order to obtain basal values and to decrease variability due to differences in nutrient absorption rates between animals (Davinder and Naugler, 2013). Concentrations of TGs in the serum of horses fed either diet were comparable to those reported in an equine study using corn oil in exercising horses (O’Connor et al., 2007) and are within the normal reference range for horses (Dugat et al., 2010). Others have reported that horses consuming SB oil have decreased serum TGs compared to those consuming a control diet, and the authors suggested this was due to increased activity of lipoprotein lipase (LPL) which led to faster clearance of TGs from the blood (Orme et al., 1997; Geelen et al., 1999). Perhaps absorption of SB in the current experiment stimulated more LPL activity, explaining the tendency for horses consuming SB to have decreased serum TGs. Serum cholesterol concentrations were greater in the current study than those reported in horses in light to medium work supplemented with vegetable oils (Geelen et al., 1999; O’Connor et al., 2007), but similar to values reported for horses at maintenance (Siciliano and Wood, 1993).

Kronfeld et al. (2004) reported that digestibility of fat in the horse should be near 100%, with the assumption that fat is completely digested in the small intestine. Certainly, many factors impact fat digestion, including DM intake, total fat intake, and fat source. Meyer et al. (1997) reported that preileal fat digestibility in horses fed a diet with 1 g added fat/kg BW was between 73% and 86% and concluded that any undigested fat would enter the cecum. There were LCFAs in the cecal fluid of horses from both treatment groups in the current study, thus indicating that digestion and absorption of fat in the small intestine are not complete when total dietary fat is approximately 4%. Increased concentration of LCFAs in the cecum of horses consuming CSFAs compared to SB at hour 2 is most likely due to less efficient absorption of CSFAs in the small intestine. Dissociation of Ca from the LCFAs may have been incomplete (Palmquist and Jenkins, 1980), thus adversely affecting digestion. Additionally, it is conceivable that differences in fatty acid composition affected digestibility, whereby greater amounts of palmitic acid in CSFAs compared to SB may have decreased prececal digestion (Doreau and Chilliard, 1997). At hour 4, dilution of LCFAs by other substrates entering the cecum may have occurred, resulting in no differences in LCFAs concentrations after hour 2. Also, retention time of LCFAs associated with concentrate entering the cecum would be minimal; therefore, LCFAs would likely leave the cecum quickly. This would further explain no differences between treatments after hour 2.

In ruminants, unsaturated LCFAs depress fiber fermentation more than saturated LCFAs (Jenkins, 1993). Because cecal unsaturated LCFAs were greater at hour 2 in horses consuming CSFAs compared with horses consuming SB, fiber fermentation may have been temporarily depressed. This hypothesis is further validated by decreased total cecal VFAs concentration at hour 2 in horses consuming CSFAs compared to horses consuming SB. However, because differences in cecal unsaturated LCFA and VFA concentrations were only noted at hour 2, it is unlikely that any deleterious effects of CSFAs on fermentation were sustained. In the end, there were no differences in NDF and ADF digestibilities between horses consuming either diet.

## SUMMARY

There is interest in increasing energy density in diets of performance horses. Increasing starch has the potential to elicit hindgut disturbances and increase excitability, both of which are undesirable. Recently, interest has shifted to increasing dietary fat as it does not cause cecal acidosis and has been shown to decrease reactivity; however, the amount of added fat in a concentrate is limited by palatability and pellet quality. CSFAs can provide a means to add more fat prior to pelleting, but the effects of CSFAs in horses have not been reported. CSFAs may not be absorbed in the foregut with the same efficiency as SB oil; however, there are no deleterious effects on total tract digestibility of fiber in the diet. Further research is warranted to determine if greater dietary inclusion rates of CSFAs would yield the same results. Based on the results of the current study, it appears that CSFAs can be included at 4% added fat in the concentrate of horses with similar effects on nutrient digestibility to a similar inclusion of SB oil.
